# Hypothalamic expression of *Peg3* gene is associated with maternal care differences between SM/J and LG/J mouse strains

**DOI:** 10.1002/brb3.58

**Published:** 2012-07

**Authors:** Silvana Chiavegatto, Bruno Sauce, Guilherme Ambar, James M Cheverud, Andrea C Peripato

**Affiliations:** 1 Department of Pharmacology, Biomedical Sciences Institute, University of Sao PauloSao Paulo, SP, Brazil; 2 Department and Institute of Psychiatry, University of Sao Paulo Medical SchoolSao Paulo, SP, Brazil; 3 National Institute for Developmental Psychiatry for Childhood and Adolescence (INCT-CNPq)Brazil; 4 Department of Genetics and Evolution, Biological Science and Health Center, Federal University of Sao CarlosSao Carlos, SP, Brazil; 5 Department of Anatomy and Neurobiology, Washington University School of Medicine63110, St. Louis, Missouri; 6 Department of Biosciences, Federal University of Sao PauloSantos, SP, Brazil

**Keywords:** Chromosome, epigenetic, *FosB*, gene expression, gene variation, hypothalamus, imprinting, maternal behavior, *Oxt*, QTL

## Abstract

Maternal care is essential in mammals, and variations in the environment provided by mothers may directly influence the viability of newborns and emotional behavior later in life. A previous study investigated genetic variations associated with maternal care in an intercross of LG/J and SM/J inbred mouse strains and identified two single-locus QTLs (quantitative trait loci). Here, we selected three candidate genes located within these QTLs intervals; *Oxt* on chromosome 2, and *FosB* and *Peg3* on chromosome 7 and tested their association with maternal care. LG/J females showed impaired postpartum nest building and pup retrieval, a one-day delay in milk ejection, reduced exploratory activity, and higher anxiety-like behavior when compared to SM/J females. The nucleotide sequences of *Oxt* and *FosB* were similar between strains, as were their hypothalamic expression levels. Conversely, *Peg3* nucleotide sequences showed four nonsynonymous replacement substitutions on LG/J dams, T11062G, G13744A, A13808G, and G13813A, and a 30 base pair (10 aa) in tandem repeat in the coding region with three copies in SM/J and five copies in LG/J. Maternal care impaired LG/J mothers express 37% lower *Peg3* mRNA levels in the hypothalamus on the second postpartum day. We also found an association of the Peg3 repeat-variant and poor maternal care in F_2_ heterozygote females derived from a LG/J × SM/J intercross. These results may suggest that the maternally imprinted *Peg3* gene is responsible for the single-locus QTL on chromosome 7 that has been shown to influence maternal care in these strains. Furthermore, these data provide additional support for an epigenetic regulation of maternal behavior.

## Introduction

Maternal care is one of the most important factors affecting offspring development, growth, and survival in mammals. After conception, murine females behave in ways that ensure offspring viability through weaning. Females usually build a nest to receive their pups and maintain it following delivery in order to keep pups warm (Lynch 1994) and protected against predators. Immediately following delivery, females must provide milk to guarantee offspring survival (Silver 1995), groom the pups, and protect them from intruders (Peripato et al. 2002). These postpartum behaviors are triggered by hormonal changes during late pregnancy and also by the presence of pups after delivery (Mayer and Rosenblatt 1987). The environment provided by mothers may also influence the emotional development of their offspring (Francis and Meaney 1999; Caspi and Moffitt 2006).

Therefore, the identification of genes that modulate maternal care is critical for an understanding of the behavioral and physiological factors underlying offspring survival, growth, and emotional behavior later in life (Lee et al. 1991; Francis and Meaney 1999). Knockout gene technology has been used to identify single genes affecting maternal care in rodents, and each of these genes are active in the CNS (central nervous system), particularly in the hypothalamus (Brown et al. 1996; Thomas and Palmiter 1997; Lefebvre et al. 1998; Lucas et al. 1998; Li et al. 1999; Collins et al. 2004). However, because maternal care is a complex trait, it is expected that several genes and the interactions between them may modulate maternal behavior. Moreover, natural variants that occur at multiple loci may contribute to differences in maternal care observed between dams.

To investigate the genetic basis of maternal care, we applied forward genetics using statistical methods (Boake et al. 2002). An intercross of LG/J and SM/J inbred mouse strains performed by Peripato et al. (2002) uncovered the genetic architecture of maternal care, including two single QTLs (chromosomes 2 and 7) and 23 epistatically interacting regions. Here, we screened the main effect regions, the QTLs at chromosome 2 and 7*,* and examined three candidate genes within these QTL intervals for their association with maternal care: *Oxt* (*oxytocin*) on chromosome 2, *FosB* (*FBJ osteosarcoma oncogene B*), and *Peg3* (*paternally expressed gene 3*) on chromosome 7.

The *Oxt* gene has a strong effect on a variety of behaviors. It participates in dependence and tolerance (Argiolas and Gessa 1991), melancholy and depression (Meynen et al. 2007; Scantamburlo et al. 2007), social recognition (Ferguson et al. 2001), anxiety (Marazziti et al. 2007), and emotional expression (Tops et al. 2007). The oxytocin peptide also acts directly on maternal care (Richard et al. 1991). The release of oxytocin during delivery triggers maternal behavior in rodents (Pedersen et al. 1982). Oxytocin-deficient female mice fail to provide milk to offspring (Nishimori et al. 1996; Young et al. 1996), although they display otherwise normal maternal behaviors. Oxytocin may also have a role in motivating the mother to retrieve her pups (Pedersen et al. 2006), and deficits in this hormone can lead to deficient offspring care (Collins et al. 2004).

The *FosB* gene belongs to the *fos* gene family known as immediate early genes (Herschman 1991; Yen et al. 1991) and is induced rapidly in specific brain regions in response to various stimuli. This induction is temporary and returns rapidly to basal levels after being triggered (Nestler et al. 1999). *FosB* has been associated with addictive behavior (Hiroi et al. 1997), response to hormones (Lin et al. 2003), and social behaviors, including pair bonding (Curtis and Wang 2003) and maternal care (Brown et al. 1996). In fact, *FosB* was the first gene described in mice to be related to maternal care (Brown et al. 1996). *FosB* knockout females fail to retrieve their pups and do not crouch over the nest.

*Peg3* encodes a zinc-finger protein (Kuroiwa et al. 1996) and is highly expressed in brain regions crucial for maternal behavior, including the medial preoptic area of the hypothalamus, the medial amygdala, the bed nucleus of the stria terminalis, the hippocampus, and olfactory bulb (Li et al. 1999). *Peg3* is a paternally expressed imprinted gene that promotes cell survival, in contrast to p53-mediated apoptosis (Relaix et al. 1998, 2000). *Peg3* affects body temperature regulation, feeding behavior, and obesity in mice (Curley et al. 2005), and its disruption can lead to aberrant maternal behavior. *Peg3*-deficient mice are viable but are smaller than wild type, and females do not build nests, fail to retrieve pups, and have lactation problems, resulting in the death of their progeny (Li et al. 1999).

We found the same maternal behavior abnormalities reported for *Oxt*-, *FosB*-*,* and *Peg3*-deficient females (Brown et al. 1996; Nishimori et al. 1996; Young et al. 1996; Li et al. 1999) to be segregated in the intercross of LG/J and SM/J inbred mouse strains (Peripato et al. 2002). Because we have previously identified these genes as positional candidates for main effect QTLs, we here investigate an association between maternal behaviors in SM/J and LG/J postpartum females and two properties of these candidate genes, DNA sequence variation and hypothalamic mRNA expression. We also tested for an effect of the *Peg3* gene in/del variant on maternal care in F_2_ females derived from a LG/J and SM/J intercross.

## Materials and Methods

### Animals

Breeding pairs of the SM/J and LG/J inbred strains (see Hrbek et al. 2006 for details on the history of these strains) were obtained from Jackson Laboratories (Bar Harbor, ME), and colonies were maintained in our facilities at the Federal University of Sao Carlos (Sao Carlos, SP, Brazil). In this study, we used the second generation of both mouse strains born in our facility. At seven weeks of age, SM/J and LG/J females were randomly mated with males of their own strain in a paired mating system. Males were removed from the breeding cage at least one week before females gave birth, at the point when the female was determined to be pregnant. Animals were fed ad libitum with Nuvilab CR1/Nuvital (Colombo, PR, Brazil) and maintained at a constant temperature of 21 ± 1°C with 12-h light/dark cycles (lights on at 6 a.m.). For the purposes of the association study of *Peg3* gene variation and maternal performance, we collected DNA from F_2_ progeny of the LG/J and SM/J intercross (Peripato et al. 2002). It is worth noting that in addition to the affected maternal care and prepulse inhibition (Samocha et al. 2010), other traits known to be affected by genetic variation in populations of LG/J and SM/J intercrosses include growth (Cheverud et al. 1996; Kramer et al. 1998; Vaughn et al. 1999), bone length (Norgard et al. 2008), obesity (Cheverud et al. 2001; Ehrich et al. 2005), and litter size (Peripato et al. 2004). Experiments were carried out in accordance with the Guidelines for the Care and Use of Mammals in Neuroscience and Behavioral Research (ILAR, Washington, D.C.), and the protocol was approved by the Ethics Committee of the Federal University of Sao Carlos (Brazil).

### Maternal performance

Maternal performance was scored as previously described (Peripato et al. 2002). A total of 30 SM/J and 23 LG/J primi-parous females were monitored daily from pregnancy detection to seven days following delivery. All procedures were conducted between 8 a.m. and 12 p.m. Litter size was scored on the day of birth, and survival was monitored daily for the first week. The maternal features investigated included nest building before and after delivery, milk provision, and pup retrieval. Two observers using the same criteria analyzed each of these features.

Nest building was determined by the presence of a nest and its quality. On the first day of pregnancy, a pressed cotton square was added to the cage. Pre- and postpartum nest building was analyzed daily by measuring nest height and scoring it as good (heights above 2.5 cm and shredded cotton in a structured way) or poor (shallow nests without cotton). Milk provision was indirectly evaluated by the presence or absence of milk in the stomach of each pup and was monitored once daily for seven days.

Females usually perform protective behavior in response to external stimuli and bring the pups back to their nest if they are removed. Thus, we scored pup retrieval on the first day postpartum by removing offspring from the nest and relocating them randomly in the cage. We monitored pup retrieval for 6 min. Females that failed to gather at least one pup in this period were considered as impaired mothers.

Anxiety-like behavior was assessed in 10 SM/J and 10 LG/J mothers on the fourth day following delivery using an elevated plus maze (EPM) according to the procedures described in Lister (1987), with some modifications. The cross-shaped apparatus consisted of two open arms (30 × 5 × 0.25 cm) arranged in opposite directions and two closed arms with acrylic transparent walls (30 × 5 × 15 cm). The cross fit into a base raised 38.5 cm above the floor. Each animal was placed at the center of the cross with its head facing an open arm, and their movements were recorded for 5 min with a video camera. The frequency and time spent in the open and closed arms, as well as the transitions between arms, were quantified.

The forced-swim (FS) test was performed in 10 SM/J and 10 LG/J mothers as described by Porsolt et al. (1977). On the sixth day following delivery, females were picked up by the tail and placed individually in a glass cylinder (40-cm deep by 20 cm in diameter) filled with water (19.5 cm) at 24°C, and their movements were video recorded for 6 min. Fresh water was replaced after each animal was tested. The amount of time animals spent immobile or swimming was recorded for the final 4 min of the test.

### Candidate genes

Two individual regions associated with maternal care on chromosomes 2 (confidence region between 72 and 108 cM) and 7 (confidence region between 0 and 14 cM) have been previously described (Peripato et al. 2002). We selected three candidate genes, *Oxt, FosB*, and *Peg3*, all of which have been previously shown to be associated with maternal care and are located within the defined chromosomal regions. These genes were sequenced and their hypothalamic expression analyzed in SM/J and LG/J dams.

#### Sequencing and microsatellite amplification

**Figure 1:**
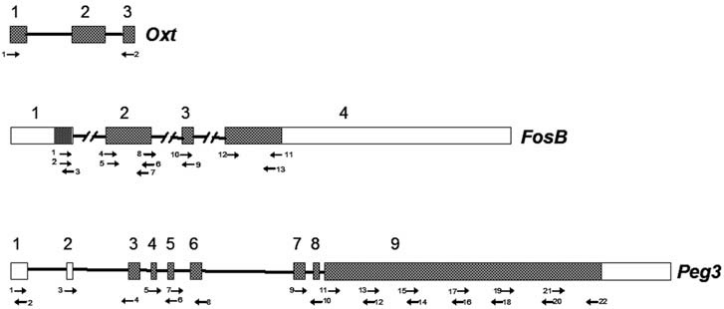
*Oxt, FosB,* and *Peg3* gene representations. Arrows indicate primer positions.

DNA was extracted from liver tissues of SM/J and LG/J females using the DNA QIAamp Tissue kit (QIAgen, Inc., Hilden, Germany). *Oxt* is located on chromosome 2 73.5 cM from the centromeric region. This gene consists of three exons and has a total length of 721 bp (Fig. 1). The exons code for a large precursor protein, which is subsequently cleaved into the following three distinct peptides: a signal peptide, the hormone oxytocin, and a membrane protein, neurophysin, which performs the intracellular transport of oxytocin (Hara et al. 1990). We designed a pair of primers to amplify the full-length *Oxt* gene (Table 1). *FosB* and *Peg3* lie in the proximal region of chromosome 7, at 9.56 cM and 3.89 cM, respectively. The *FosB* candidate gene is approximately 5000-bp long and consists of four exons (Fig. 1). We therefore designed 13 primers, which allowed for 10 unique amplification combinations (Fig. 1; Table 1). *Peg3* is 15.5-kb long and consists of nine exons (Fig. 1). We designed 11 primer pairs to amplify all exons (see Fig. 1; Table 1). DNA was sequenced using a DYEnamic ET Terminator kit (GE-Healthcare, Buckinghamshire, UK), and DNA fragments were resolved using a MegaBACE 1000 DNA Analysis System (GE-Healthcare).

**Table 1:** Forward and reverse primers sequences for *Oxt, FosB,* and *Peg3* genes for DNA sequencing and PCR amplification

Gene	Exon	Primer	Forward 5′ > 3′	Primer	Reverse 5′ > 3′	Product size (bp)
*Oxt*	1, 2, and 3	Oxte1F	CCATCACCTACAGCGGATCTCAGAC	Oxte3–2R	AAGGCAGACTCAGGGTCGCAGGC	725
*FosB*	1	FosBe1F	AGCAGCGCACTTTGGAGACGTGTC	FosBe1R	TAAAACTTACCTGGGAGGCGGCGG	234
*FosB*	1	FosBe1(2)F	CCCCGTGAAACCGACAGAGCCTG	FosBe1R	TAAAACTTACCTGGGAGGCGGCGG	367
*FosB*	2	FosBe2F	GTCTCTCTTATCTCTCTTGGGCGT	FosBe2R	GTCTCTTCTCGGGGTCTTCTAGGC	379
*FosB*	2	FosBe2F	GTCTCTCTTATCTCTCTTGGGCGT	FosBe2(2)R	ACTGTACAAACTGAGCCCACATCCC	448
*FosB*	2	FosBe2(2)F	GACACACACATCCACACCCGCTCA	FosBe2R	GTCTCTTCTCGGGGTCTTCTAGGC	429
*FosB*	2	FosBe2(2)F	GACACACACATCCACACCCGCTCA	FosBe2(2)R	ACTGTACAAACTGAGCCCACATCCC	498
*FosB*	2 and 3	FosBe2–3F	ATGCCAGGAACCAGCTACTCAACCCC	FosBe2–3R	AAGTGGGGAACAGTCGAAAGTAAGTGGG	890
*FosB*	3	FosBe3F	CAGAAGAAGAAGAAAAGCGAAGGG	FosBe2–3R	AAGTGGGGAACAGTCGAAAGTAAGTGGG	266
*FosB*	3 and 4	FosBe3F	CAGAAGAAGAAGAAAAGCGAAGGG	FosBe3–4R	TTCCTTCTCTTTTTGCAGCTCGGC	1490
*FosB*	4	FosBe4F	GCCGAGCTGCAAAAAGAGAAGGAA	FosBe4R	CAGAGCAAGAAGGGAGGGCGAGTT	387
*Peg3*	1	Peg3e1F	AGGACGAGCATCGGAGGAGAAGC	Peg3e1R	AGCACAGCACTCTACGCACACACC	103
*Peg3*	2 and 3	Peg3e2F	GACAACTGGCAAGAGGAAGACTAGG	Peg3e3R	GGTACATCTTGAAACTCTCCAACGG	919
*Peg3*	4 and 5	Peg3e4F	TGAACAGTGACGACGACATGAGCC	Peg3e5R	CTTCTGGGATTCCTGGTGTATGGC	733
*Peg3*	5 and 6	Peg3e5F	AAGCCCCCTGATACCTTTCTTCCTT	Peg3e6R	CCATGGAACGTCTGTCATCATCTC	1063
*Peg3*	7 and 8	Peg3e7F	GATGCCGAGTCATACCAGAATGTTG	Peg3e8R	GCTTGGGTAGGCAGTTCTCTTGGA	891
*Peg3*	9(1)	Peg3e9(1)F	GTGGGATCTGTGAGGACGAGTCTT	Peg3e9(1)R	GCCACGCTATGAATAAAGGACTC	635
*Peg3*	9(2)	Peg3e9(2)F	GAGTCCTTTATTCATAGCGTGGC	Peg3e9(2)R	GGGAATTTCAGCTTGTCATTAGGG	854
*Peg3*	9(3)	Peg3e9(3)F	CCCTAATGACAAGCTGAAATTCCC	Peg3e9(3)R	TCTTGGGCATAACTGGTCTGAGGG	761
*Peg3*	9(4)	Peg3e9(4)F	GTGACCCTCAGACCAGTTATGCCC	Peg3e9(4)R	CAACAGTGCAATTTCTCCTTGGTC	890
*Peg3*	9(5)	Peg3e9(5)F	GACGCTTTCATCGCTCTGTTGCCC	Peg3e9(5)R	CCTCTGGCTCTTCGACGTCTTCC	809
*Peg3*	9(6)	Peg3e9(6)F	GGAAGACGTCGAAGAGCCAGAGG	Peg3e9(6)R	AGTGTGAGAATTCTGGTGTCTGGC	356

We also designed primers Peg3e9(4)F and Peg3e9(4)R (Table 1) to flank a region in *Peg3* exon 9 in which we detected a large in/del that distinguishes the SM/J and LG/J strains (described in the Results section). We used these primers to amplify this region from 240 F_2_ females derived from the LG/J and SM/J intercross to investigate a correlation between this specific *Peg3* polymorphism and maternal performance based on offspring survival, and to investigate for possible association of the different genotypes in this region and their maternal performance.

#### Hypothalamic Oxt, FosB, and Peg3 expression

Female SM/J and LG/J mice (*n*= 13 each) were carefully removed from the nest on the second postpartum day and sacrificed by decapitation between 8 a.m. and 12 p.m. We chose the second postpartum day, when mother–infant interaction is totally established and the probability of pups from maternally impaired mothers still being alive is higher. The whole hypothalamus was immediately dissected on ice and stored in RNA*later*® (Ambion, Austin, TX) in microtubes. Samples were kept at room temperature (RT) for 1 h and stored at –80°C until use. The hypothalamus was removed from the RNA*later*®, immersed in TRIzol (Invitrogen, Sao Paolo, SP, Brazil) and homogenized (Polytron PT10/35-Brinkmann, Westbury, NY) for 30 sec at maximum speed. Total RNA was isolated using the manufacturer's protocol and quantified in a spectrophotometer (NanoDrop® ND-1000, Wilmington, DE). RNA purity was assessed with the 260/280 nm ratio, and its integrity was assessed on a 1% agarose gel. Total RNA was treated with DNase I (Invitrogen) (1 U/μg of RNA, at 37°C for 20 min), and 2 μg from both experimental groups were simultaneously reverse transcribed using oligo(dT) primers and SuperScript™ III reverse transcriptase (Invitrogen) in a final volume of 20 μL.

Quantitative analyses of the *Oxt, FosB*, and *Peg3* transcript levels was carried out in a Rotor-Gene 3000 (Corbett Research, Concord, Australia) using SYBR green, according to Ambar and Chiavegatto (2009). Optimal conditions for PCR were obtained using a five-point, twofold dilution curve analysis using RG-3000 (Corbett Research) software for each transcript. Each PCR reaction contained the equivalent of 12.5 ng of reverse-transcribed, DNase-treated RNA, 200 nM of each specific primer, SYBR Green PCR Master Mix (Applied Biosystems, Foster City, CA), and RNase-free water to a 20 μL final volume. cDNA samples from both groups were assayed in the same run, in triplicate, in 0.1-mL microtubes. Samples without cDNA templates and samples with RNA (no reverse transcription) were included as negative controls in all experiments. A dissociation curve was performed following each run to further confirm product specificity and the absence of primer dimers. Real-time PCR efficiencies for each reaction varied from 99% to 109%, and the correlation coefficient was not lower than 0.99. Real-time data were collected and analyzed in Excel. The relative amount of *Oxt, FosB*, and *Peg3* transcripts between SM/J and LG/J samples was calculated according to Vandesompele et al. (2002), as previously described (Chiavegatto et al. 2010). The following genes were analyzed as controls: *cyclophilin A* (*peptidylprolyl isomerase A: Ppia*), *hypoxanthine guanine phosphoribosyl transferase 1* (*Hprt1*), and *beta-actin* (*Actb)*. Primers for candidate and control genes were designed in different exons when possible (Table 2), according to criteria detailed elsewhere (Bibancos et al. 2007).

**Table 2:** Forward and reverse primers sequences for hypothalamic RNA expression

Gene	NCBI reference sequence	Forward 5′ > 3′	Reverse 5′ > 3′	Product size (bp)
*Oxt*	NM_011025	GCTGCCAGGAGGAGAACTAC	ATGGGGAACGAAGGAAGC	204
*FosB*	NM_008036	CCTTCAACCAGCACAACCA	ACGGTTCCTGCACTTAGCTG	159
*Peg3*	NM_008817	AGTCCAGCTTGCCGAAGAT	CTCAGGCATGGGTTTGAGAC	112
*Ppia*	NM_008907	AATGCTGGACCAAACACAAA	CCTTCTTTCACCTTCCCAAA	101
*Hprt1*	NM_013556	TGTTGTTGGATATGCCCTTG	GCGCTCATCTTAGGCTTTGT	106
*Actb*	NM_007393	GTGGGAATGGGTCAGAAGG	GGTCATCTTTTCACGGTTGG	228

### Statistical analysis

Behavioral data were compared using a two-tailed Student's *t* test, and the associations among nominal variables were tested by cross tabulation using a Pearson χ^2^ test and ϕ coefficient in SYSTAT 10.0.

The base-calling quality for *Oxt, FosB,* and *Peg3* was visually inspected using Chromas software (http://www.technelysium.com.au/chromas.html. Forward and reverse sequences for each gene region were manually evaluated, aligned, and compared between SM/J and LG/J strains. These analyses were also performed using the BioEdit Sequence Alignment Editor (Hall 1999). The GenBank (NCBI) accession numbers for SM/J and LG/J gene sequences are, respectively, HQ679943 and HQ679944 (*Oxt*)*,* HQ679939 and HQ679940 (*FosB*), HQ679941 and HQ679942 (*Peg3*). The association between maternal care (absence or presence) and genotypes for the exon 9 *Peg3* marker in F_2_ females was investigated using standard analysis of variance (ANOVA)-–General Linear Model in SAS, v.9.0 (SAS, 2004).

Transcript quantities were tested for a normal distribution (Kolmogorov–Smirnov test) and compared using a two-tailed Student's *t* test (GraphPad InStat® version 3.05, San Diego, CA). Data were expressed as mean ± standard error of the mean (SEM) or median and range. Differences were considered statistically significant when *P* < 0.05.

## Results

### LG/J females have poorer maternal performance when compared to SM/J females

**Figure 2:**
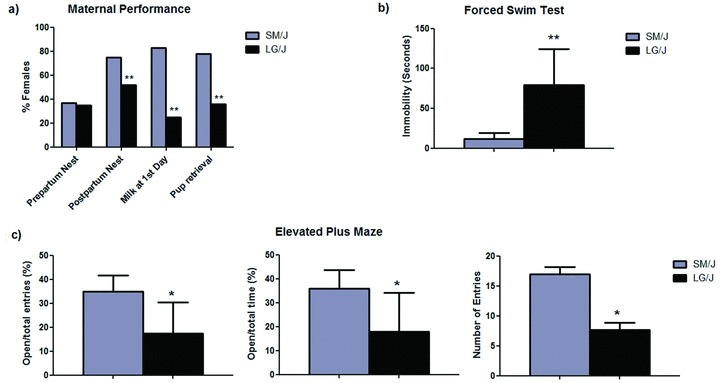
Maternal attributes of SM/J and LG/J mice inbred females. (a) Maternal performance: prepartum nest indicates the percentage of females with good quality, prepartum nests; postpartum nest indicates the percentage of females with good quality, postpartum nests; milk at first day is the percentage of females that had pups with milk in their stomachs on the first day after delivery, indicating lactation; pup retrieval indicates the percentage of females exhibiting defensive maternal behavior at postpartum day 1, returning pups to the nest during the 6 min following the disturbance (*n*= 30 SM/J; 23 LG/J). (b) Forced swim test activity: time spent immobile in the final 4 min (*n*= 10 each group on sixth day after delivery). Means ± SEM; **P < 0.01. (c) Elevated plus maze activity: relative number of entries and time spent on open arms in relation to closed arms in 5 min (*n*= 10 each group on fourth day after delivery). Means ± SEM; *P < 0.05.

SM/J and LG/J females display distinctive levels of maternal performance (Fig. 2). Although both females usually built a prepartum nest and maintained it after giving birth, only SM/J mothers displayed a more sophisticated postpartum nest (ϕ= 0.38, *P* < 0.05). Pups from SM/J mothers had milk in their stomachs as soon as the first day of life, which was not observed in pups delivered by LG/J dams, which only presented milk from the second day forward (ϕ= 0.57, *P* < 0.001). SM/J females groomed their pups and retrieved them after nest disturbance more frequently than LG/J mothers on first day after birth (ϕ= 0.43, *P* < 0.01). The survival rate for animals born to SM/J mothers was 72% while only 35% of the pups born to LG/J dams were viable after one week (*P* < 0.01).

In the FS test, LG/J females spent more time in an immobile or floating position than SM/J female mice (*P* < 0.01). In the EPM test, LG/J females spent less time and made fewer forays into the open arms of the apparatus when compared to SM/J mothers (*P* < 0.05 for both parameters). LG/J females also had a lower absolute frequency of entries into either of the arms (*P* < 0.05).

We found a correlation between pup retrieval behavior and immobility in the FS test (–0.53; *P* < 0.05). The performance in the EPM test was also correlated with pup retrieval (0.70 for time and 0.62 for entries in the open arms; *P* < 0.01 for both).

### Sequencing of candidate genes and *Peg3* sequence variations

**Figure 3:**
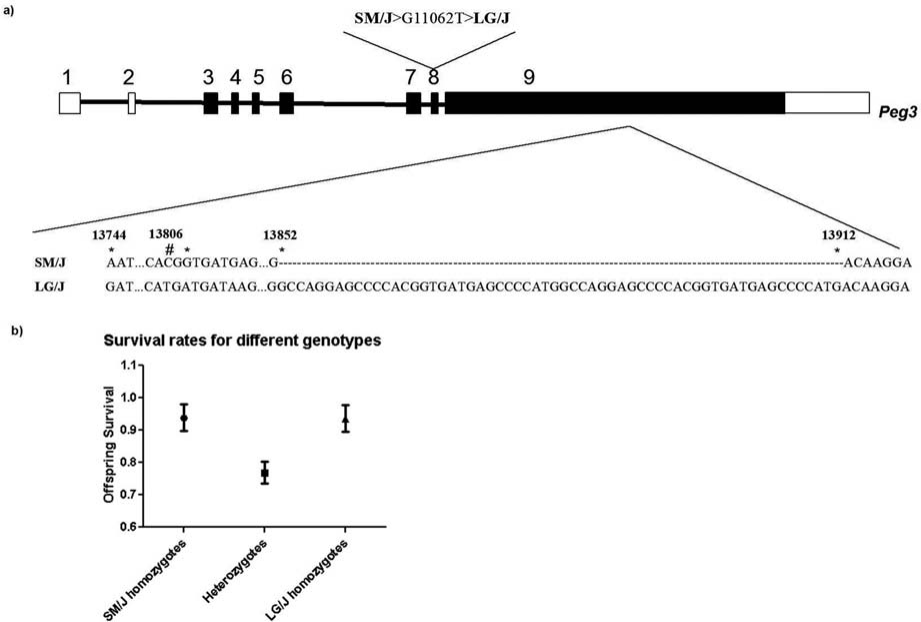
Sequencing and genotype variations in *Peg3*. (a) Representation of the nine exons of the *Peg3* mouse gene. Positions of the *Peg3* sequence variations in SM/J and LG/J strains are shown in the expansions. * represents a non-silent variation, # represents a silent variation, and – represents a deletion. Variation on exon 8 involves amino acid change. Numbers above symbol indicate substitution or deletion positions. (b) Genotypes for *Peg3* insertion variation in F_2_ females derived from a LG/J × SM/J intercross and their maternal performance.

*Oxt, FosB,* and *Peg3* sequences in SM/J and LG/J mice showed high similarity to sequences from other *Mus musculus* strains in the Mouse Genome Database (MGB–NIH). *Oxt* showed no sequence variation between SM/J and LG/J. When we compared *FosB* gene sequences, we found only a G insertion in intron 1 in LG/J, but not in the SM/J strains. However, when we compared the *Peg3* sequence between SM/J and LG/J mouse strains, we found several relevant differences. The LG/J *Peg3* sequence has four replacement substitutions, one on exon 8, T11062G (Leu>Arg), and the others on exon 9, namely G13744A (Asp>Asn), A13808G (Asp>Gly), and A13813G (Lys>Glu). There was also a silent substitution, T13806C (His) and a 30-bp (10 aa) tandem repeat in the coding region of *Peg3*. The LG/J strain showed five copies of this repeat, but only three copies were observed in the SM/J strain (13852Δ13912) (Fig. 3).

### *Peg3* gene variation in F_2_ dams is correlated with offspring survival

We investigated whether the *Peg3* tandem repeat occurring three times in SM/J and five times in LG/J was associated with maternal failure based on offspring survival. The length of the polymorphism in exon 9 of the *Peg3* gene was examined using PCR, and this allowed for genotyping of F_2_ individuals derived from LG/J and SM/J intercrosses, which segregated these alleles. We found that F_2_ female heterozygotes for this allele showed, on average, impaired offspring survival when compared to females with either homozygote genotype (Fig. 3; *P* < 0.01).

### *Peg3* hypothalamic expression is lower in LG/J females, but Oxt and FosB expression levels are not affected

**Figure 4:**
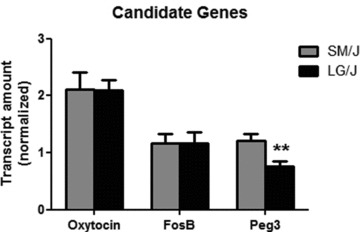
*Oxt, FosB*, and *Peg3* transcript levels in the hypothalamus of female mice on the second day postpartum. Chromosome 2 QTL candidate gene, *Oxt*, shows no significant differences in expression between SM/J and LG/J females. mRNA transcript levels for the chromosome 7 QTL candidate gene, *FosB*, are similar between strains. The third candidate gene, *Peg3*, shows 37% lower transcript levels in the hypothalamus of LG/J dams than in SM/J dams. Quantities were normalized against the geometric mean of *Ppia* and *Actb* mRNA levels (*n*= 13 each group). Means ± SEM; *P < 0.05.

The levels of *Oxt* and *FosB* transcripts in the hypothalamus of SM/J and LG/J females on the second postpartum day were similar between strains (*P* > 0.05) (Fig. 4). Alternatively, *Peg3* transcripts were lower in the hypothalamus of female LG/J mice on the second day postpartum when compared to SM/J females (–37.4%, *P* < 0.01) (Fig. 4). In these samples, *Ppia* and *Actb* showed the most stable levels (M = 0.91, geNorm *applet*) and were therefore used to normalize transcript levels for the candidate genes. Transcript levels for all tested control genes were similar for both strains (*Ppia*: 0.43 ± 0.06 vs. 0.41 ± 0.04; *Actb*: 0.28 ± 0.07 vs. 0.25 ± 0.04; and *Hprt1*: 0.61 ± 0.05 vs. 0.59 ± 0.05; *n*= 13 per group; SM/J vs. LG/J, respectively, *P* > 0.05).

## Discussion

Caring for, feeding, and protecting pups after birth are the primary maternal behaviors responsible for offspring survival and growth in rodents (Lee et al. 1991). Nest building is a common maternal care that keeps pups together and confined in the first days of life allowing their temperature maintenance (Lynch 1994; Gaskill et al. 2011) and the close contact with their mother (Fleming et al. 1999). In the present study, we demonstrate that female mice of both SM/J and LG/J strains generally build a nest before labor and maintain it after delivery. However, with respect to the quality of these nests in the postpartum period, SM/J females displayed more sophisticated nests using the material provided, in contrast to shallow and less elaborate nests built by LG/J dams. Generally, mothers show some response to sensorial stimuli after birth coming from pups (Rosenblatt 1975; Weber and Olsson 2008) and the presence of pups may reflect in postpartum nest building, as we may see through the higher complexity in the genetic architecture of postpartum than prepartum nest-building behavior (B. Sauce, R. A. de Brito and A. C. Peripato, unpubl. data). Therefore, based on nest quality criteria, LG/J mothers showed impaired nest-building behavior.

Milk provision is essential in mammals, so it is crucial that females provide food immediately after pups are born (Silver 1995). Most SM/J females exhibited milk ejection at the first day postpartum, but LG/J females had a one-day delay in milk letdown. This delay observed in LG/J dams does not seem to be due to pups’ suckling impairment, since we tested the ability to suck milk from a dropper in some one-day LG/J pups. Food deprivation caused by a deficit of milk in the first day of life may impact offspring growth and predispose animals to develop obesity in adulthood (“thrifty phenotype”), when they have access to food ad libitum (Hales and Barker 2001). We found exactly this pattern in animals of LG/J × SM/J intercross, in which the absence of milk in their stomach at the first day of life was associated to animals’ tendency to be heavier due to fat deposition (C. P. Góes, B. Sauce and A. C. Peripato, unpubl. data). The larger size and fat deposition observed in LG/J animals is also reported for *Peg3*-deficient animals (Curley et al. 2005).

Litter protection is another important maternal attribute that is divergent between SM/J and LG/J mothers. Most SM/J females rescued their pups immediately after pups were relocated outside the nest. In contrast, LG/J mothers did not display this behavior in the 6 min tested. Occasionally, when each LG/J cage was reexamined hours later, pups remained unretrieved. Because protective maternal posture is modified by the act of pups suckling at the nipples (Erskine et al. 2004) and most LG/J females did not have milk on the first day after delivery, pup retrieval may be a maternal behavior delayed in LG/J females as well.

Additional postpartum behaviors tested in SM/J and LG/J females included those associated with anxiety and depression-like behaviors. It is important to note that mothers’ manipulation for EPM and FS testing (fourth and sixth postpartum day) did not influence the outcome of maternal performance, because the establishment of poorer maternal behavior of LG/J females when compared to SM/J was established sooner at the second postnatal day. In both tests, LG/J females diverged from SM/J females, exhibiting more anxious or fearful behaviors and displaying lower exploratory drive and increased immobility in the FS test. Depressed or anxious mothers with low motivation may be impaired in maternal care and neglect their newborns (Field 1998; Champagne et al. 2003). However, we do not rule out the possibility that anxiety and depression-like behaviors are independent features of the phenotypic profiles of LG/J versus SM/J females.

Together, these findings clearly indicate that LG/J females are impaired in each of the investigated maternal attributes when compared to SM/J females and further indicate that LG/J litters have compromised viability.

*Oxt* is a gene associated with milk ejection (Nishimori et al. 1996; Young et al. 1996) and pup retrieval (Pedersen et al. 2006), and is thus a strong candidate gene. Moreover, it is positioned in the confidence interval of the chromosome 2 QTL previously associated with maternal care (Peripato et al. 2002). Although milk ejection and pup retrieval were compromised in LG/J dams, we did not find sequence variations in *Oxt* between SM/J and LG/J mice, nor did we observe differences in hypothalamic expression on the second postpartum day. Thus, our data do not support the participation of *Oxt* in the previously reported QTL. However, a contribution of the oxytocin peptide in the impaired maternal behavior of LG/J mothers cannot be ruled out given that we did not investigate the expression on different time points, nor posttranscriptional modifications that could lead to reduced peptide levels, or alternatively, alter the status of oxytocin receptors in the mammary glands or brains of these animals.

A role for *FosB* gene in maternal behavior was suggested by studies using mice lacking this gene (Brown et al. 1996). *FosB* knockout females show deficits in pup retrieval and poor nest-building behavior, which we similarly observed in LG/J females. *FosB* is located within the single QTL interval reported for chromosome 7 in a LG/J × SM/J intercross (Peripato et al. 2002). The sequencing analyses performed in the present study revealed no variation in *FosB* exons between the two strains. The single insertion found in intron 1 in LG/J animals did not impact *FosB* expression levels in the hypothalamus, making it unlikely that *FosB* participates in the observed variation in maternal care between SM/J and LG/J females.

There is, however, another strong candidate gene in the QTL on chromosome 7, identified by Peripato et al. (2002). This gene is *Peg3*, which also has previously been shown to have a direct association with maternal care. *Peg3* knockout females show similar types of abnormal maternal care as *FosB* knockout females, in addition to lactation problems, anxious behavior, and lower locomotor activity (Li et al. 1999; Champagne et al. 2009). LG/J mothers share many of these behavioral and physiological traits. Comparison of the *Peg3* sequences between SM/J and LG/J revealed four nonsynonymous substitutions and an increased number of 30-bp (10 aa) tandem repeats in exon 9. This sequence repeats three times in the SM/J strain and five times in the LG/J strain. These extra copies in LG/J *Peg3* exon 9 may impact the protein structure and consequently its function. These findings raised the possibility that these gene variations may be associated with the differing maternal phenotypes observed between SM/J and LG/J dams. We focused on the exon 9 *Peg3* in/del variation to further investigate an association of genotype and maternal care as judged by offspring survival. In order to address this question, we analyzed F_2_ females derived from a LG/J × SM/J intercross. We found that heterozygous F_2_ females showed, on average, impaired maternal care when compared to homozygous females. These results are in line with our previous findings on the underdominant nature of the QTL at the proximal end of chromosome 7; that is, heterozygote females provide poor maternal care when compared to the parental genotypes (homozygote for SM/J or LG/J alleles) (Peripato et al. 2002).

Notably, *Peg3* transcripts were also lower in the hypothalamus of the maternal care impaired LG/J females when compared to SM/J females on the second postpartum day. These results may suggest that the *Peg3* gene polymorphism found in LG/J dams negatively impacts *Peg3* hypothalamic expression. Lower levels of *Peg3* transcripts in LG/J females and the heterozygote genotype for the LG/J allele in F_2_ females were similarly correlated with poor maternal care.

*Peg3*, as the name implies, is a paternally expressed gene and shows a functional nonequivalence for allelic expression based on parent-of-origin. Imprinted genes have been associated with fetal growth, placental function, and behaviors. Although maternal expression is associated with fetal growth (Tycko and Morison 2002), paternal expression often favors placental development (Reik et al. 2003), and both modulate neurodevelopment, even in postnatal life (Davies et al. 2005; Gregg et al. 2010a, b). Recent studies in mice have revealed the increased complexity behind the putative roles for imprinted genes in the brain by showing their spatial and temporal regulation (Gregg et al. 2010a, b). Although parental effects in the developing and adult brain differ, studies have found that in the adult hypothalamus, approximately 70% of imprinted autosomal genes are preferentially expressed through the paternal allele. Accordingly, *Peg3*, similar to several other paternally expressed genes in the hypothalamus, is associated with maternal care (Lefebvre et al. 1998; Li et al. 1999; Curley et al. 2004; Champagne et al. 2009), thereby demonstrating the role of epigenetics in mammalian behavior. It is noteworthy that the underdominance for *Peg3* in F_2_ females is an atypical effect for a parent-of-origin gene. Although the heterozygote effect is not in accordance with what would be expected given the imprinted, paternal expression of *Peg3*, this dominance pattern was also previously observed in the ovine callipyge gene (Cockett et al. 1996).

Mutant, postpartum *Peg3* females show reduced immunoreactivity for oxytocin in the hypothalamus when compared with wild-type females, which could explain their impairments in lactation (Li et al. 1999). In the present study, although hypothalamic *Oxt* transcript levels were not reduced in LG/J females when compared with those of SM/J mothers, we cannot exclude the possibility of a posttranscriptional effect in this peptide hormone, an effect possibly induced by reduced levels of *Peg3*.

In summary, the *Peg3* gene maps to the chromosomal region where we previously identified a QTL affecting maternal performance in an intercross of LG/J × SM/J inbred mice. Analysis of the *Peg3* gene sequence in LG/J and SM/J female mice revealed several variations leading to amino acid substitutions, as well as a large insertion (10 aa) in a coding region, resulting in a different number of tandem repeats between the strains. Furthermore, *Peg3* gene expression in the hypothalamus of LG/J postpartum females is remarkably lower than in SM/J dams. Interestingly, LG/J mothers exhibit many of the same maternal care impairments observed in *Peg3* knockout females, including deficits in pup retrieval, milk ejection, locomotion, and an increase in anxiety-like behaviors. F_2_ females derived from the LG/J × SM/J intercross also show an association between *Peg3* genotype and maternal performance, thus increasing the likelihood of the participation of this gene in maternal behavior. *Peg3* has a high level of sequence similarity between mice and humans (Kim et al. 1997, 2000), is also imprinted in humans (Murphy et al. 2001), and has a similar protein expression pattern in the brains of both species (Kim et al. 1997), suggesting conserved functions. Thus, our results further implicate this paternally expressed, maternally imprinted gene in inappropriate maternal behavior. These data also support future studies to investigate human variants and to study associations between *Peg3* and nurturing dysfunctions.
